# The Immunohistochemical Evaluation of Solid Pseudopapillary Tumors of the Pancreas and Pancreatic Neuroendocrine Tumors Reveals ERO1Lβ as a New Biomarker

**DOI:** 10.1097/MD.0000000000002509

**Published:** 2016-01-15

**Authors:** Junjie Xie, Yi Zhu, Hao Chen, Minmin Shi, Jiangning Gu, Jiaqiang Zhang, Baiyong Shen, Xiaxing Deng, Xi Zhan, Chenghong Peng

**Affiliations:** From the Department of Surgery, Ruijin Hospital, Shanghai Jiao Tong University School of Medicine, Shanghai (JX, HC, JG, JZ, BS, XD, XZ, CP); Department of Surgery, The Second Affiliated Hospital of Zhejiang University, Hangzhou (YZ); and Research Institute of Digestive Surgery, Ruijin Hospital, Shanghai Jiao Tong University School of Medicine, Shanghai, P.R. China (MS).

## Abstract

Solid pseudopapillary tumor of the pancreas (SPTP) is a class of low-grade malignant tumors that carry a favorable prognosis after surgery. Our group has reported that dysfunctions in the endoplasmic reticulum (ER) protein processing pathway may play a role in tumor development. However, alterations of this pathway in other pancreatic tumors had not been well investigated. In this study, we collected 35 SPTP and pancreatic neuroendocrine tumor (PNET) specimens and described the clinicopathological features of them. We performed immunohistochemistry (IHC) for 6 representative proteins (ERO1Lβ, TRAM1, GRP94, BIP, P4HB, and PDIA4) involved in the ER pathway in both SPTP and PNET specimens. We compared the IHC scoring results of tumors and matched normal pancreas tissues and demonstrated that these proteins were downregulated in SPTP specimens. Five of these proteins (TRAM1, GRP94, BIP, P4HB, and PDIA4) did not display significant changes between PNET and normal pancreas tissue. However, ERO1Lβ was upregulated in PNET tissues compared to the normal tissues, which could be used as a pathological biomarker in the future.

## INTRODUCTION

Solid pseudopapillary tumor of the pancreas (SPTP) represents a rare type of pancreatic neoplasm that exhibits low-grade malignant potential. Patients with SPTP have a favorable prognosis with a 5-year survival rate > 85% after complete surgical resection. A minority of patients with recurrent SPTP or with liver metastasis also have good long-term survival with other therapies.^1–3^ Recently >700 cases have been well studied.^4^ However, the origin of disease, direction of differentiation, and pathogenesis remain elusive. SPTP may resemble pancreatic neuroendocrine tumor (PNET) both in histomorphology and immunophenotype. Several useful pathological biomarkers had been identified to distinguish these 2 tumors, such as β-catanin, E-cadherin, and P504s.^5–7^ However, we still need to find some new proteins or pathways to better understand the differences of these 2 diseases.

The researchers focused on the disease at a molecular level found that the Wnt/b-catenin, Hedgehog, and androgen receptor signaling pathways, as well as genes involved in epithelial-mesenchymal transition, were activated in SPTP by a microarray.^8^ Deregulated expression of cell cycle-associated proteins was also identified in SPTP.^9^ Our previous proteomics studies had shown that ∼37 proteins belonged to the endoplasmic reticulum protein processing pathway were downregulated in SPTP tissues compared to matched normal pancreas tissues.^10^

In this study, 6 representative proteins (ERO1Lβ, TRAM1, GRP94, BIP, P4HB, PDIA4) belonging to the endoplasmic reticulum protein processing pathway were chosen. Our goal was to determine if these 6 proteins were also downregulated in PNET, which may help us to determine if this pathway change was unique to SPTP. As a result, we determined that 5 of these 6 proteins (TRAM1, GRP94, BIP, P4HB, PDIA4) did not change obviously in PNET tissues compared to matched normal pancreas tissues. However, the expression of ERO1Lβ was significantly upregulated in PNET tissues, which could serve as a useful pathological biomarker in the future.

## MATERIALS AND METHODS

### Sample Collection

A total of 35 surgically resected SPTP and 35 surgically resected PNET specimens were collected at the Department of General Surgery, Institute of Digestive Surgery, Ruijin Hospital, Shanghai JiaoTong University Medical School from 2005 to 2011. All the specimens were obtained at the time of surgery and then fixed in 10% formaldehyde and processed routinely for paraffin embedding. All samples were obtained from patients with informed consent and with approval of the institutional ethics committee. All cases were diagnosed on the basis of clinical, imaging, histopathologic, and immunohistochemical investigations. Sections were cut into 4 mm sections and were stained with hematoxylin and eosin. The representative areas of each tumor selected on H&E stained slides, and the corresponding normal tissue were arrayed with a tissue cylinder 1 mm in diameter.

### Immunohistochemistry (IHC)

The SPTP (n = 35), PNET (n = 35), and matched normal tissue microarrays were used to test the expression patterns and intensities for 6 proteins (ERO1Lβ, TRAM1, GRP94, BIP, P4HB, PDIA4) by IHC. The following primary antibodies were used: anti-TRAM1 rabbit polyclonal, 1:100 dilution, anti-PDIA4 rabbit polyclonal, 1:100 dilution; anti-GRP94 mouse monoclonal, 1:200 dilution, anti-BIP rabbit polyclonal, 1:50 dilution, anti-P4HB mouse monoclonal, 1:400 dilution, and anti-ERO1Lβ rabbit polyclonal, 1:100 dilution. All antibodies were obtained from LifeSpan Biosciences. After deparaffinization and rehydration, antigen retrieval was performed. The details of IHC process were described in our previous article.^10^

### Score Grading

All slides were evaluated by 2 pathologists and the disagreements were resolved by discussions. The final IHC scores included 4 degrees of immunoreactivity: strong (3+), moderate (2+), mild (1+), and negative (0). The details of method of IHC score grading had been described in Remmele's article.^11^

### Statistical Method

The statistical differences in the IHC scores were analyzed by a paired *t* test. *P* < 0.05 was considered to indicate a statistically significant difference. Statistical analysis was performed using the Statistical Program for Social Sciences (SPSS) software 17.0 (SPSS Inc., Chicago, IL).

## RESULTS

### Clinical and Pathological Features of SPTPs and PNETs

SPTP samples from 35 patients in this study consisted of 27 female patients and 8 male patients. Their ages ranged from 11- to 58-year old, and the median age was 31-year old. The tumor size ranged from 2 to 8 cm, and the median size was 4.0 cm. CD10 was expressed in 6 cases. With the exception of 1 case, CD99 showed a paranuclear “dot-like” pattern in all SPTP tissues.^12,13^ The expression of E-cadherin was lost in all specimens, and β-catanin was mainly localized in the nucleus of the tumor cells. Insulin was expressed in 2 cases. The 35 PNET patients from whom tissues were collected consisted of 17 female and 18 male patients. Their ages ranged from 11- to 58-years-old, although the median age was 50-year old. The tumor size ranged from 2 to 8 cm and the median size was 2.1 cm. CD10 was expressed in 8 cases. CD99, E-cadherin, and β-catanin were expressed in most of the 35 cases and was mainly localized to the membrane and cytoplasm of the cells. Insulin was expressed in 30 cases. The details were shown in Table 1 .

**TABLE 1:**
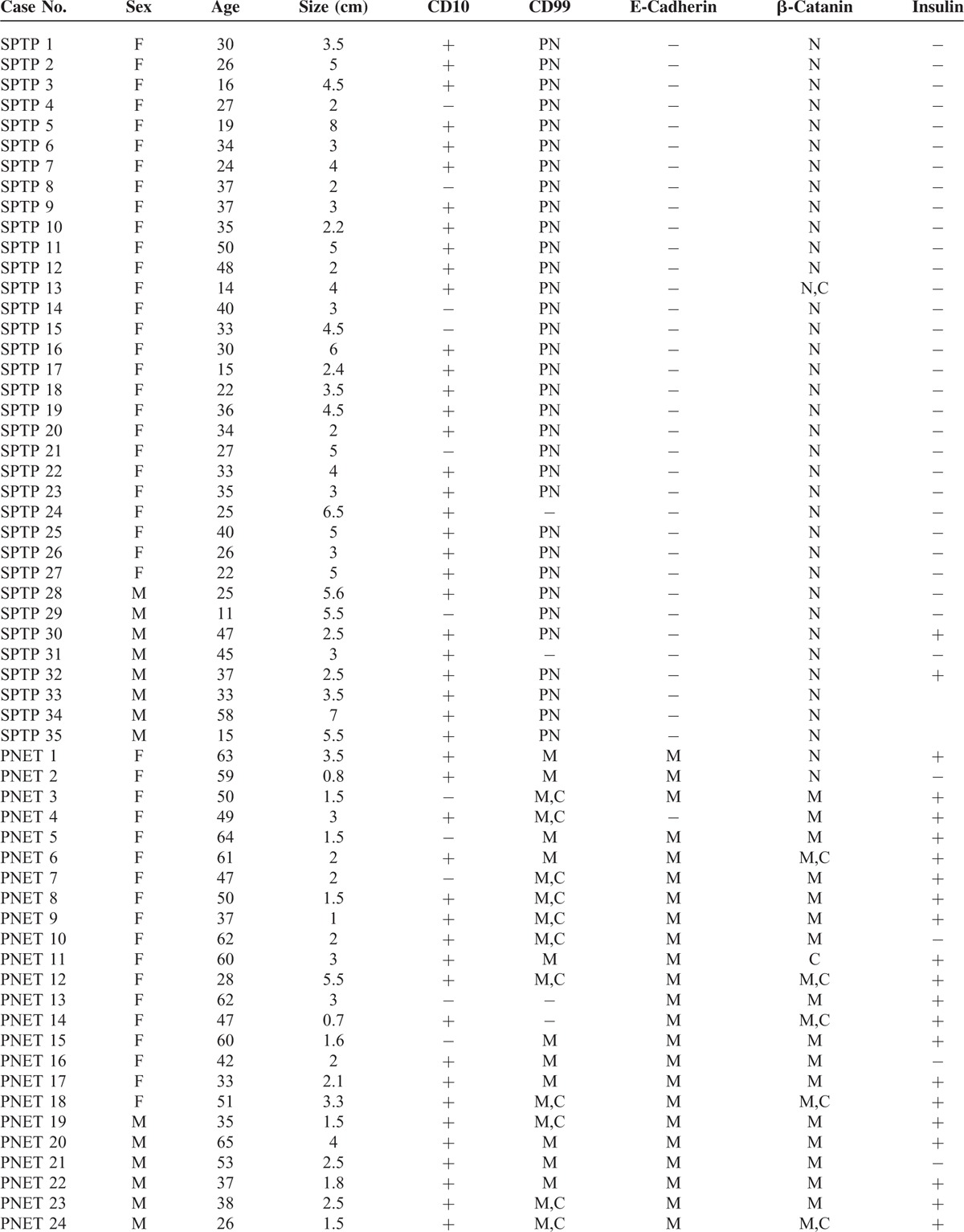
Clinical and Pathological Features of 35 SPTP and PNET Patients

**TABLE 1 (Continued):**
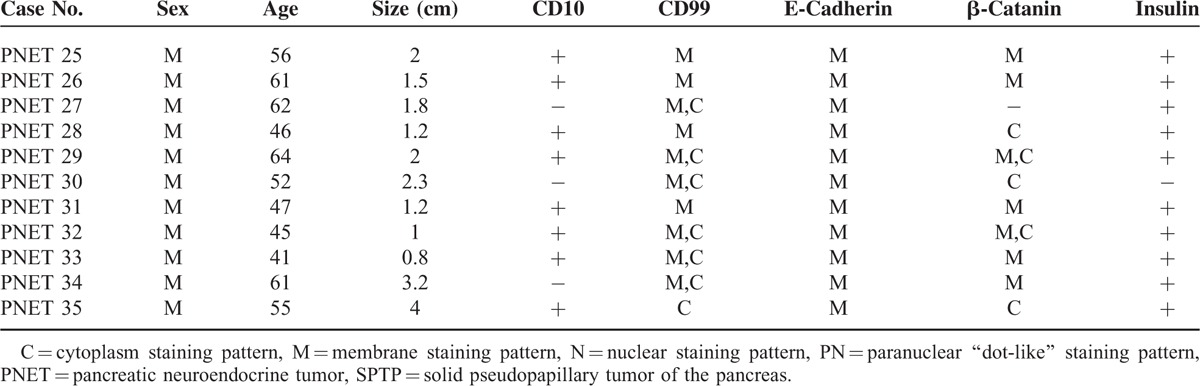
Clinical and Pathological Features of 35 SPTP and PNET Patients

### Immunohistochemistry Findings in SPTP and PNET Specimens

**FIGURE 1:**
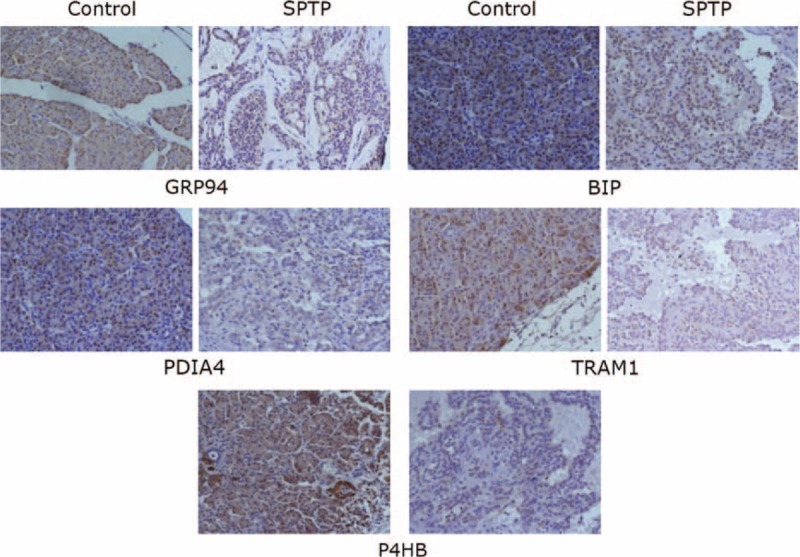
Immunohistochemistry image for markers associated with the endoplasmic reticulum protein processing pathway in individual SPTP tissues and corresponding normal pancreas tissues from the same patient (350×). TRAM1, GRP94, BIP, P4HB, and PDIA4 expression was confirmed in SPTP specimens. The IHC protocol is described in the *Materials and Methods*. The staining scoring details are shown in Table 2. IHC = immunohistochemistry, SPTP = solid pseudopapillary tumor of the pancreas.

**FIGURE 2:**
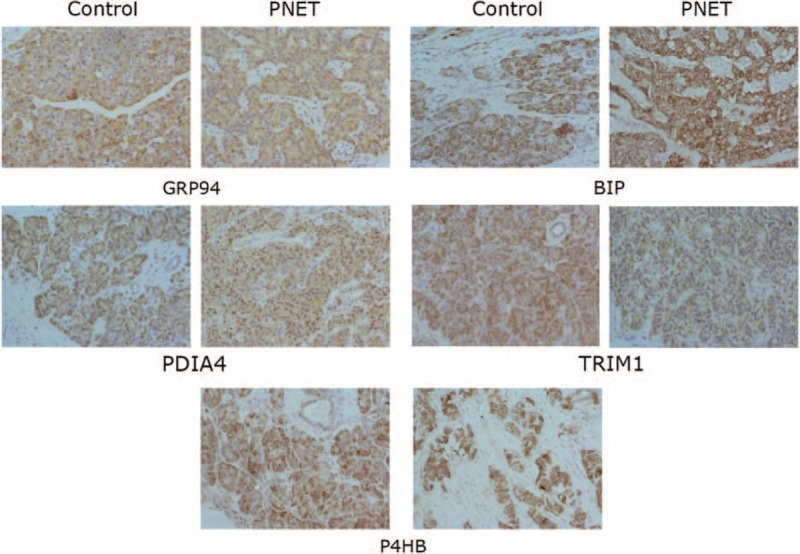
Immunohistochemistry image for markers associated with the endoplasmic reticulum protein processing pathway in individual PNET tissues and corresponding normal pancreas tissues from the same patient (350×). TRAM1, GRP94, BIP, P4HB, and PDIA4 expression was confirmed in SPTP specimens. The IHC protocol is described in the *Materials and Methods*. The staining scoring details are shown in Table 3. IHC = immunohistochemistry, PNET = pancreatic neuroendocrine tumor, SPTP = solid pseudopapillary tumor of the pancreas.

**FIGURE 3:**
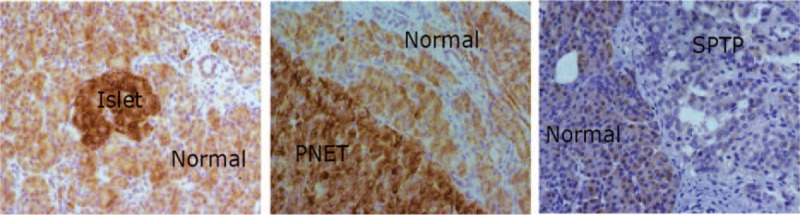
Representative immunohistochemistry image for ERO1Lβ in islet tissue, PNET tissue, and SPTP tissue (350×). The left panel shows islet and normal pancreas, the middle panel shows PNET and normal pancreas, and the right panel shows SPTP and normal pancreas. PNET = pancreatic neuroendocrine tumor, SPTP = solid pseudopapillary tumor of the pancreas.

In our previous proteomic study, 6 proteins (ERO1Lβ, TRAM1, GRP94, BIP, P4HB, PDIA4) (Table 2) involved in the ER protein processing pathway were found to be downregulated in SPTP tissues compared to the normal pancreas tissues, whereas these proteins were mainly localized to the cytoplasmic fraction of the normal pancreas and tumor tissues. We previously demonstrated that these proteins were expressed in a limited number SPTP specimens. Therefore, we expanded our study to a sample size to 35 SPTP specimens. Furthermore, we wanted to determine if the expression of these 6 proteins were downregulated in PNET tissues compared to matched normal pancreas tissues using immunohistochemistry methods. Immunohistochemical findings for normal pancreas, SPTP and PNET tissues are summarized in Tables 3 and 4, and examples are illustrated in Figures 1 and 2. All 6 markers showed higher IHC staining scores (*P* value < 0.05) in normal pancreatic tissues compared to the matched SPTP tissues. We found that 5 of these proteins (TRAM1, GRP94, BIP, P4HB, PDIA4) did not show significant differences in the majority of PNET tissues compared to the matched normal tissues. However, ERO1Lβ was differentially expressed in the normal pancreas compared to PNET tissues; the intensity of immunoexpression was different in exocrine and endocrine components. From the IHC results, we determined that ERO1Lβ was upregulated in PNET tissues and mainly localized to the cytoplasm, which resembled the Langerhans islets. However, ERO1Lβ was downregulated in SPTP tissues compared to normal tissues (Figure 3).

**TABLE 2:**
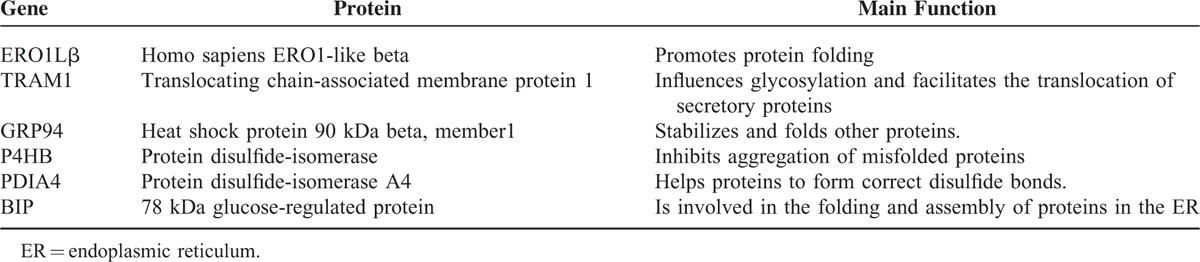
The Main Function of 6 Proteins (ERO1Lβ, TRAM1, GRP94, BIP, P4HB, PDIA4) Involved in the Endoplasmic Reticulum Protein Processing Pathway

**TABLE 3:**
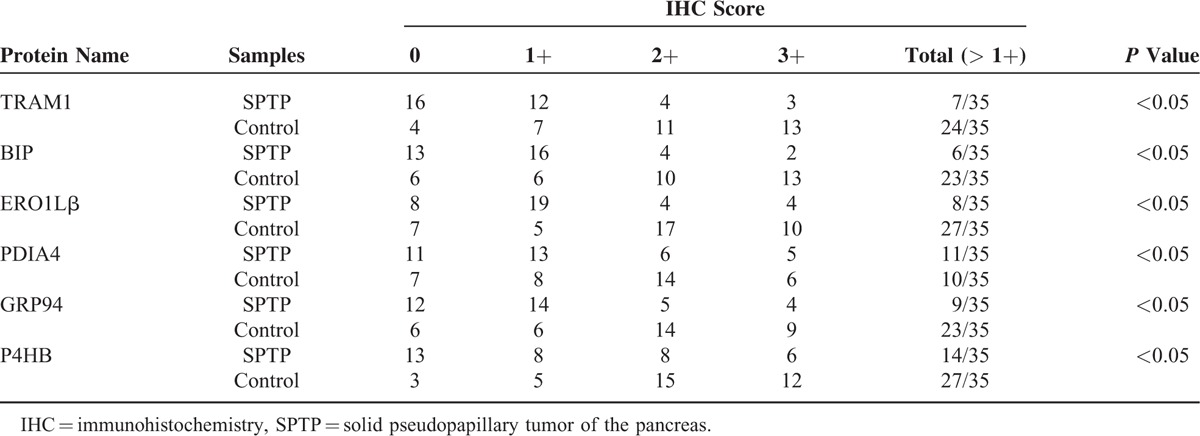
IHC Staining Scores in SPTP and Matched Normal Pancreas Tissues (control) for 6 Markers Associated With the Endoplasmic Reticulum Protein Processing Pathway (an IHC Score of 9–12 Was Considered a Strong Immunoreactivity (3+); 5–8, Moderate (2+); 1–4, Weak (1+); and 0, Negative)

**TABLE 4:**
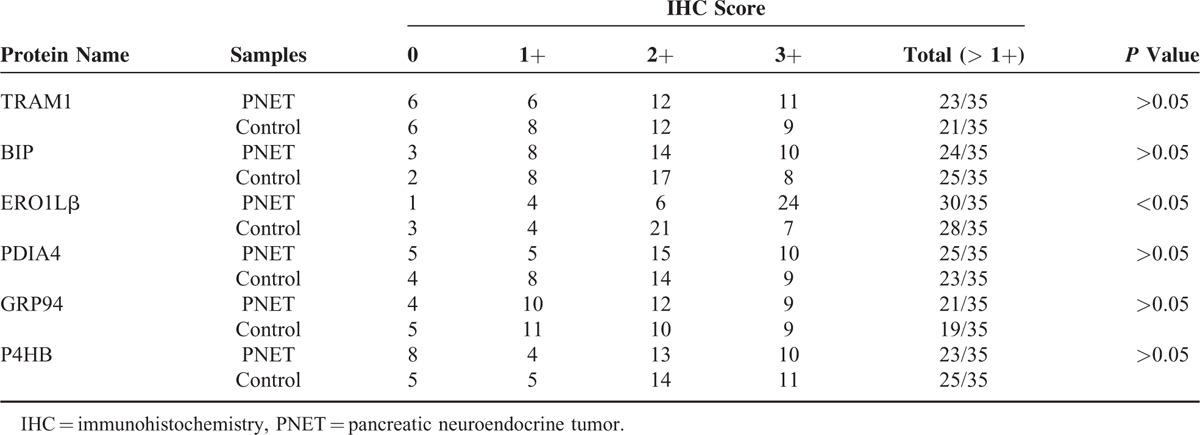
IHC Staining Scores in PNET and Matched Normal Pancreas Tissues (control) for 6 Markers Associated With the Endoplasmic Reticulum Protein Processing Pathway (an IHC Score of 9–12 Was Considered a Strong Immunoreactivity (3+); 5–8, Moderate(2+); 1–4, Weak(1+); and 0, Negative)

## DISCUSSION

In this study, we collected the clinical and pathological data of 35 pairs SPTP and PNET specimens. We then tested the expression levels of 6 proteins involved in the ER protein processing pathway.

The clinical and pathological data of these patients showed that SPTP mainly occurred in young women, whereas there were no gender differences in PNET patients, although patients with PNET were on average older than SPTP patients. The average tumor sizes for patients with SPTP were larger than tumors in patients with PNET. PNET patients tend to exhibit obvious symptoms, which would likely lead to an earlier detection and smaller tumor size at diagnosis. The pathological biomarkers detected validated that a paranuclear “dot-like pattern” of CD99 may be a useful biomarker to distinguish SPTP from PNET,^12,13^ whereas the nuclear accumulation of β-catenin and loss of E-cadherin expression was obvious in SPTP specimens.

Our goal was to determine if changes in the ER protein processing pathway are unique to SPTP patients (compared to the PNET patients). Six proteins (ERO1Lβ, TRAM1, GRP94, BIP, P4HB, PDIA4) involved in the ER protein processing pathway were downregulated in SPTP specimens, but not PNET specimens. We selected to examine these proteins in our study for the following reasons: (1) these 6 proteins belong to the different functional arms of this pathway, (2) these showed the most significant differences as far as fold changes in our proteomic results, and (3) these had been shown to have important biological functions (Table 2).^14–19^ Our results suggest that the ER protein processing pathway is possibly involved in SPTP tumorigenesis, but does not necessarily contribute to the development of PNETs. Our previous proteomic study identified >30 proteins belonging to this pathway that were downregulated in SPTP tissues. This finding suggests that there is a possible relationship between ER stress and SPTP. Although these 6 proteins do not represent the entire pathway, their expression pattern gives some clues for further investigations to elucidate the origins of SPTPs. ERO1Lβ is a pancreas-specific disulfide oxidase that is known to be upregulated in response to ER stress and to promote protein folding in pancreatic β cells. We found that high expression of ERO1Lβ was concentrated in pancreas tissues, mainly in the pancreatic islets. This protein can promote insulin biogenesis and glucose homeostasis. Deregulation of ERO1Lβ can contribute to the pathogenesis of diabetes mellitus.^20^ However, this molecule has not been previously implicated in the development of PNETs, which mainly originate from islet cells. In our study, we found that the expression level of ERO1Lβ was higher in most PNET specimens compared to the normal pancreas, which may contribute to the synthesis of insulin. In SPTP specimens, reductions in the expression of ERO1Lβ and our other proteins of interest may indicate that the rate of mature protein synthesis is impaired in SPTPs. Furthermore, because ERO1β expression differs between normal pancreas, PNET, and SPTP specimens, it could be a useful pathological marker to differentiate PNETs from SPTPs.

Our work has several limitations. First, the statistical results could not avoid potential information bias because the 2 pathologists could not totally blind to the histopathological diagnosis when they faced to the IHC results. Next, we could not conclude that the protein involved in ER pathway was a unique change in SPTP because we did not focus on other pancreatic tumor such as acinar carcinoma. Furthermore, we did not associate the pathway with the prognosis of SPTP because of the follow-up was short, which will be calculated in the future. Our goal was to clarify if these changes happened in PNET, which was an extension of our proteomic results.
